# Potential clinical value of catheters impregnated with antimicrobials for the prevention of infections associated with peritoneal dialysis

**DOI:** 10.1080/17434440.2023.2205587

**Published:** 2023-05-03

**Authors:** Hari Dukka, Maarten W. Taal, Roger Bayston

**Affiliations:** a Consultant Nephrologist, Department of Renal Medicine, University Hospitals of Derby and Burton NHS Foundation Trust, Derby, UK; b Professor of Medicine, Centre for Kidney Research and Innovation, Academic Unit for Translational Medical Sciences, School of Medicine, University of Nottingham, Nottingham, UK; c Emeritus Professor, Academic Unit for Injury, Repair and Inflammation Sciences, School of Medicine, University of Nottingham, Nottingham, UK

**Keywords:** Antimicrobials, catheter, impregnation, peritonitis, peritoneal dialysis

## Abstract

**Introduction:**

Peritoneal dialysis (PD) is a widely used dialysis modality, which offers the advantage of being a home therapy but is associated with a risk of potentially serious infections, including exit site infection, catheter tunnel infection, and peritonitis that may result in morbidity, technique failure, and increased mortality. Catheters impregnated with antimicrobials hold promise as a novel technique to reduce PD associated infections.

**Areas Covered:**

We describe PD modalities, catheters, technique, complications, and the microbiology of associated infections, as well as standard measures to reduce the risk of infection. A novel technique for the impregnation of silicone devices with antimicrobial agents has been used to produce antimicrobial impregnated ventricular shunt catheters with proven clinical efficacy that have now been adopted as the standard of care to reduce neurosurgical infections. Using the same technology, we have developed PD and urinary catheters impregnated with sparfloxacin, triclosan, and rifampicin. Safety and tolerability have been demonstrated in urinary catheters, and a similar study is planned in PD catheters.

**Expert Opinion:**

Catheters impregnated with antimicrobials offer a simple technique to reduce PD associated infections and thereby enable more people to enjoy the advantages of PD. Clinical trials are needed to establish efficacy.

## Lay summary

Peritoneal dialysis (PD) is a type of treatment for kidney failure. To perform PD, a silicone tube is placed in the abdomen and the other end exits through the skin. Fluid is run into the abdomen via the tube and then drained out again after 1–12 hours. This process is repeated multiple times per day. Toxins and other waste chemicals normally removed by the kidneys enter the fluid, while it is in the abdomen and are then removed from the body when the fluid is drained out. In this way, PD partially replaces kidney function. Sometimes bacteria get into the tube, and this can cause serious infections in the abdomen. At present, measures available to prevent PD tube infections include careful hygiene when handling the tube, application of antibiotic creams or ointments to the exit site or treatment with antibiotics at the time of medical procedures that may increase infection risk. Despite these measures, peritonitis (abdominal infection) is one of the most common causes of people having to stop PD and change to another form of dialysis that involves direct filtration of the blood (hemodialysis). Frequent use of antibiotics may also cause the bacteria that cause peritonitis to become resistant to antibiotics. There is, therefore, an urgent need to develop new ways to prevent PD tube infections. Tubes have been used in patients who have a particular type of brain surgery with antibiotics introduced into the material that the tube is made from, and in these patients, the risk of infection has been reduced by 60–80%. The same technology is also being tested for urine tubes that are placed in the bladder and tubes used for PD. These urine tubes and PD tubes need further testing to establish safety and effectiveness. Though our experience with them leads us to expect that they are safe, the authorities that control new drugs and devices require us to show this beyond doubt before they can be introduced into routine care.

## 1. Introduction

Patients who develop end-stage kidney disease (ESKD) need renal replacement therapy (RRT) to survive. RRT includes dialysis treatment and transplantation. Dialysis can be done in-center, which involves visits to hospital or a dialysis center, or at home. Peritoneal dialysis (PD) is a form of home dialysis, which has been in use since the 1950s. To enable PD, a catheter is inserted into the abdomen (pouch of Douglas), and dialysis fluid (containing electrolytes and glucose or an alternative osmotic substance) is infused into the peritoneal cavity. The peritoneum acts as a semi-permeable membrane and allows removal of toxic substances and excess water. There are several advantages of dialyzing at home, which include better quality of life compared to in-center hemodialysis (HD) [1]. PD also has an advantage of causing no hemodynamic compromise and better preservation of residual kidney function, which has been shown to improve patient survival [2]. PD is also relatively cost-effective due to lower staff costs and does not require large amounts of building space for delivery. Consequently, the PD patient population prevalence has been increasing considerably around the world [3]. Due to the COVID pandemic, there has been significant strain on health-care resources and in-center hemodialysis facilities, highlighting the benefits of home-based PD. There are currently estimated to be 369,000 ESKD patients receiving peritoneal dialysis worldwide, representing 11% of the global dialysis population [4].

Nevertheless, PD is associated with a substantial risk of infections such as peritonitis, catheter tunnel, and exit site infections, which are responsible for 30% to 50% of PD technique failures and the most common reason for patients being switched to HD. Moreover, PD catheter-related infections may require hospital admissions, emergency surgical removal of the catheter, and are associated with increased risk of mortality [5]. Some patients on PD may be uniquely vulnerable to infection by virtue of also receiving immunosuppressant medication and/or chemotherapy. Interventions are therefore needed to reduce the risk of catheter-related infections. In this paper, we review the basic concepts of peritoneal dialysis and propose a novel approach to reduce the risk of infection using antimicrobial impregnated catheters.

## 2. Peritoneal dialysis access

**Figure 1.**
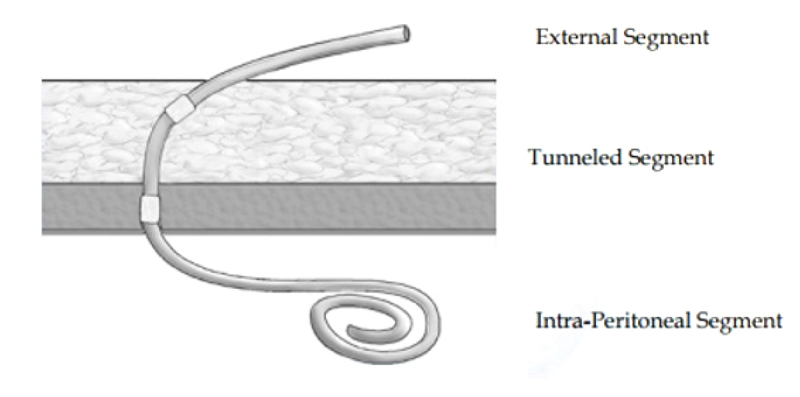
Diagram of Tenckhoff catheter placed in the abdominal cavity for peritoneal dialysis showing placement of the tip in the pelvis and transcutaneous segment. Used with permission from Kidney International (Publisher: Elsevier).

The key to successful chronic PD is a safe and permanent access to the peritoneal cavity. Tenckhoff and Schechter catheters are widely used for this purpose. The Tenckhoff catheter is a silicone tube with side holes along its intraperitoneal portion. There are usually one or two Dacron cuffs attached to the catheter, which provoke fibrous tissue growth around them, securing the catheter in place and preventing peri-catheter leakage and infection. One cuff is positioned close to the entry of catheter into the peritoneum, and an outer cuff is close to the skin exit. A significant proportion of the catheter is located transcutaneousy (Figure 1). Over the years, many modifications of the Tenckhoff catheter have appeared. Although several studies report less frequent catheter drainage failures with the use of the arcuate ‘swan neck’ catheter compared with straight catheters, there is no hard evidence that any of the modified catheters are better than the original design (one- or two-cuff) [6].

There are several methods by which a Tenckhoff catheter can be placed inside the peritoneal cavity, including a variety of open surgical techniques, laparoscopic surgery, and percutaneous insertion [7]. Surgical and laparoscopic methods usually require a general anesthetic and are mostly performed by surgeons. The advantage of these procedures is that they allow direct visualization of the peritoneal cavity and usually lead to better catheter position and relatively less risk of catheter migration. Laparoscopic techniques also allow for the catheter to be stitched into the pelvis, which avoids catheter migration and reduces the risk of inadequate dialysis. Lysis of intraabdominal adhesions can also be performed during catheter placement if present. The percutaneous technique can be performed either by a surgeon or a physician under local anesthetic. This technique is more suitable for elderly patients who may not be at high risk of complications from general anesthesia. Complications associated with PD catheter insertion include bowel perforation, more common with percutaneous technique, serous and dialysis fluid leaks, hemorrhage, and catheter malfunction due to dislocation. Infections such as peritonitis and infection around the catheter exit site may also occur and the risk of infections can be reduced by administration of pre – procedure antibiotics [8].

## 3. Dialysis fluid and PD modalities

Peritoneal dialysis involves infusion of a dialysis fluid into the peritoneal cavity via the peritoneal dialysis catheter. The fluid consists of osmotic agents, buffers, and electrolytes and is allowed to dwell in the peritoneal cavity for 1–12 hours. Metabolites and electrolytes are transferred into the dialysis fluid by diffusion and convection, and water is removed by osmosis.

Dextrose is a commonly used osmotic agent and other osmotic agents include a glucose polymer, icodextrin, which is less readily absorbed from the peritoneal cavity, amino acids, and polypeptides. Three different agents have been used as buffers to control acidosis: lactate, bicarbonate, and acetate. Dialysis solutions also contain sodium, magnesium, calcium, and chloride to prevent excessive loss of electrolytes.

Peritoneal dialysis can be performed in a continuous (continuous ambulatory peritoneal dialysis [CAPD]) or an automated form of intermittent dialysis (automated peritoneal dialysis – APD) [9]. CAPD involves multiple exchanges during the day (usually three), followed by an overnight dwell. APD uses a cycler to perform multiple overnight exchanges, resulting in multiple short dwells [10]. Variations of APD include continuous cycler peritoneal dialysis (CCPD), nightly intermittent peritoneal dialysis (NIPD), tidal peritoneal dialysis, and intermittent peritoneal dialysis. CCPD and NIPD differ from each other by the absence or presence of a daytime fluid dwell.

## 4. PD technique

**Figure 2.**
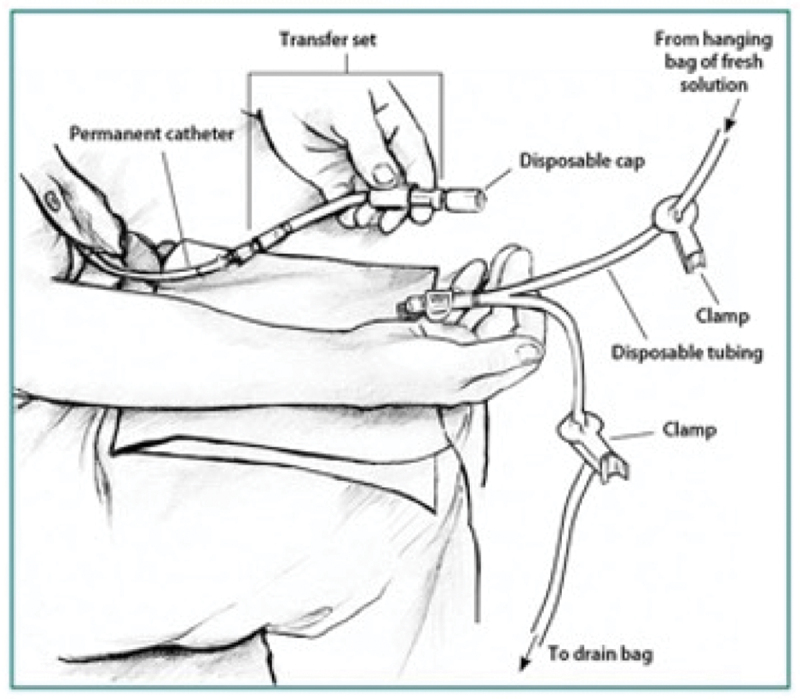
Diagram illustrating the technique for exchange of fluid during peritoneal dialysis. Used with permission from National Institute of Diabetes and Digestive and Kidney Diseases, National Institutes of Health.

PD fluid is commercially supplied in bags, which come connected to a Y-shaped giving set. The patient manually connects the short arm of the Y connector to the PD catheter. The other arm of the Y-shaped giving set is attached to an empty dialyzate bag (Figure 2). A small volume of dialysis fluid is drained directly from the new bag into the empty bag and in principle this flushes away any bacteria at the end of the catheter. This has been named the ‘flush before fill’ technique. After this, the dialysate in the peritoneal cavity from the previous exchange is drained out into the empty bag. Once this process is finished, fresh dialysis fluid is infused into the peritoneal cavity via the PD catheter after clamping the long arm of the Y connector, which leads to the bag that now contains drained dialysate. The ‘flush before fill’ technique has been shown to reduce peritonitis rates [11]. Patients are trained to follow strict hand hygiene and to follow an aseptic technique while performing exchanges to reduce the risk of infections.

## 5. Complications of PD

Though PD is a very effective treatment, there are some complications and risks to patient health. These can be classified as noninfectious and infectious complications. Noninfectious complications include dialysis fluid drainage problems, pericatheter fluid leaks, abdominal discomfort, dialysis fluid leak into the pleura and electrolyte imbalances [12]. Dialysis fluid drainage problems are often due to constipation, which can be diagnosed with an abdominal X-ray and treated with laxatives. Other drainage issues may be due to deposition of fibrin and thrombi within the catheter, which can be treated with heparin or urokinase. Catheter kinks, which can also lead to drainage problems, require replacement of the PD catheter. Abdominal discomfort may be due to increased intra-abdominal pressure and volume, and some patients develop associated gastroesophageal reflux disease. Dialysis fluid leak into the pleura can occur due to the presence of a pleuroperitoneal fistula. This usually requires discontinuation of PD and transfer to HD treatment. Hypokalaemia is very common in PD patients and requires dietary advice and potassium supplementation.

## 6. Infectious complications of peritoneal dialysis

Infectious complications of PD include exit site infections, catheter tunnel infections, and peritonitis. The PD catheter and exit site may become colonized by bacteria soon after its insertion into the peritoneum. Bacteria form a biofilm that provides protection against host defense mechanisms [13]. Any minor trauma at the exit site can lead the colonizing bacteria to cause infection. Examination of biofilms formed on PD catheters often show bacteria normally found on skin such as staphylococcus and streptococcus. Other bacteria responsible for biofilm formation are *Escherichia coli*, *Klebsiella pneumoniae*, *Pseudomonas aeruginosa, Proteus mirabilis*, and enterococci [14]. Studies have shown biofilm formation to be the cause of recurrent and relapsing peritonitis as bacteria in biofilm mode are considerably less susceptible to antibiotics [15].

### 6.1. Peritonitis

Peritonitis is a very common complication of PD and is associated with significant risk of technique failure and transfer to HD and also increased mortality. Global peritonitis rates are variable, ranging between 0.16 episodes per patient-year to 0.4 episodes per patient-year [16]. The International Society of Peritoneal Dialysis (ISPD) recommendation is to keep peritonitis rates below 0.4 episodes per patient-year. The signs and symptoms of peritonitis commonly include abdominal pain, cloudy dialysis fluid, fever, diarrhea, and vomiting. Diagnostic criteria for peritonitis are: 1) clinical signs and symptoms of peritonitis present, 2) dialysis effluent white cell count >100/µL or >0.1 × 10^9^/L (after a dwell time of at least 2 hours), with >50% polymorphonuclear leukocytes, 3) positive bacterial culture of dialysis effluent. A diagnosis is made if at least two criteria are met [16]. There are several risk factors for developing PD peritonitis, including constipation, inadequate hand hygiene, medical procedures such as colonoscopy, colposcopy, and dental work [16,17]. Any surgical procedure holds similar risks and it is recommended to drain dialysis fluid completely from peritoneum before any procedure, to avoid bacterial translocation into the dialysis fluid. Other factors associated with the risk of peritonitis are obesity, hypoalbumiemia, hypokalemia, chronic obstructive pulmonary disease, and smoking. Nephrology centers provide training to patients to enable them to perform dialysis fluid exchanges using an aseptic technique, but as the process involves manually connecting the bags to the PD catheter there remains a risk of introduction of organisms by touch contamination [17]. Being a new starter on PD is another risk factor. The risk of developing peritonitis was 55% in the first year after starting PD and 89% within the first 3 years in one study [18]. This may be due to lack of patient experience in doing PD using aseptic techniques.

### 6.2. Microbiology of PD peritonitis

Bacteria are the predominant cause of PD peritonitis. Gram-positive organisms are responsible for 30–60% of cases, while gram-negative organisms cause 15–30% of the cases. Fungi such as candida account for 3–5% of cases. Culture-negative peritonitis is also common and is found in 13–40% of the cases [19].

The commom gram-positive organisms include staphylococci, streptococci, *Corynebacterium* spp., and enterococci. Coagulase negative staphylococci (CoNS) account for most peritonitis cases followed by streptococci. The introduction of ‘flush before fill’ systems has reduced the incidence of peritonitis caused by CoNS but has had no effect on cases caused by *Staphylococcus aureus* and gram-negative bacteria [11]. Touch contamination is responsible for most cases of peritonitis caused by gram-positive organisms.

Gram negative peritonitis is usually caused by *E. coli*, *Klebsiella* spp, and *Ps aeruginosa* which may come from the bowel, skin, urinary tract, contaminated water, or contact with pets [20]. Measures introduced in most nephrology centers to stem infections caused by gram-positive organisms have led to relative increased rates of peritonitis caused by gram-negative organisms [21].

Culture-negative peritonitis accounts for 13–40% of cases. The most common reason for obtaining culture-negative peritonitis is obtaining cultures after administration of antibiotics. Infective causes for culture-negative peritonitis include Mycobacteria, fungal pathogens, Nocardia, and Legionella [16].

Fungal peritonitis is usually caused by Candida species and accounts for 3–5% of cases. Fungal peritonitis carries is a high mortality risk, and timely removal of the PD catheter is essential [16].

### 6.3. PD catheter tunnel and exit site infection

Any minor trauma to the exit site can lead to exit site infection (ESI), which can progress to catheter tunnel infection and peritonitis. ESI is diagnosed if purulent discharge is noted at the exit site with or without erythema. A scoring system for monitoring exit sites (Table 1) has been developed by pediatricians and is presented in the 2017 ISPD guideline on catheter-related infections but has not yet been validated in adult patients [22]. Risk factors for developing ESI are trauma, which is usually caused by pulling the catheter accidentally, poor exit site care, compression of the catheter by waist belt, presence of pets at the time of exit site care or dialysis exchanges, or swimming [23]. Most ESIs are caused by gram-positive organisms, such as *S. aureus* and CoNS. The rest are caused by gram-negative organisms such as pseudomonas. Fungal ESIs are very rare and usually indicate contamination, hence repeat culture is usually needed to confirm the diagnosis. ESI may lead to infection of the subcutaneous tunnel unless appropriately treated with antibiotics. Catheter tunnel infection can be diagnosed if erythema, induration, and tenderness are present on and around the catheter tunnel. Ultrasound may also be used to diagnose occult tunnel infection [22]. Catheter tunnel infection sometimes leads to peritonitis and catheter replacement is therefore usually indicated.

**Table 1.** Proposed criteria for the diagnosis of exit site infections (reference 22).

	Score		
Parameter	0	1	2
Swelling	No	Exit only (<0.5 cm)	>0.5 cm or tunnel or both
Crust	No	<0.5 cm	>0.5 cm
Redness	No	<0.5 cm	>0.5 cm
Pain	No	Slight	Severe
Drainage	No	Serous	Purulent

Infection should be assumed with a score of 4 or higher. Purulent drainage, even by itself, is sufficient to indicate infection. A score of 4 or less may or may not represent infection.

### 6.4. Treatment and prognosis of peritonitis

Peritonitis is associated with significant morbidity and mortality. The treatment of peritonitis requires intra-peritoneal (IP) antibiotics such as vancomycin, second-generation cephalosporins, or aminoglycosides. IP antibiotics are needed for at least 2 weeks and if dialysate white cell count remains greater than 100/µL after day 5 of treatment, then catheter removal is indicated [16]. Catheter removal is generally needed for fungal peritonitis, as it has a high mortality risk [16]. Management of peritonitis is usually conducted in an outpatient setting, unless a patient is septic and/or needs catheter removal. About 20% of peritonitis cases require catheter removal, which is more commonly associated with *S. aureus* and gram-negative organisms. In one study, peritonitis was associated with a 95% increase in all-cause mortality [5,24]. This is more commonly associated with *S. aureus*, gram-negative organisms, and fungal peritonitis. Catheter removal also requires transfer to HD at least on a temporary basis, which may compromise a patient’s quality of life especially if they are elderly and comorbid. Peritonitis may also lead to decreased volume of fluid removal due to change in transport status of the peritoneal membrane [25]. This may be temporary or a permanent effect and may lead to significant fluid retention in patients, which has a high risk of mortality.

## 7. Prevention of exit site infections and peritonitis

### 7.1. Exit site care and aseptic technique

Multiple strategies have been employed to prevent ESI. In some centers, patients are regularly screened for nasal carriage of *S. aureus,* and those found to be colonized are treated with mupirocin, a topical antibacterial agent. Patients are also trained and advised on daily exit site care, which includes cleaning the exit site with chlorhexidine or povidone iodine solution and application of agents such as mupirocin or gentamicin ointment [26]. The exit site should be monitored at least monthly by clinical staff to facilitate prompt identification and treatment of ESI.

Peritonitis prevention strategies include training patients on hand hygiene and appropriate methods of aseptic connection and disconnection of the dialyzate bags to the catheter [26]. In some centers, this training is repeated every 6 months. Prophylactic IP antibiotics such as vancomycin or cefuroxime are given before PD catheter insertion to reduce the risk of peritonitis. Patients should also receive prophylactic IP antibiotics before procedures such as colonoscopy, colposcopy, and cystoscopy [16]. Despite all these measures, peritonitis remains a common problem with rates exceeding the target of 0.4 episodes per patient-year in many centers.

### 7.2. Catheter coatings

Attempts to reduce exit site infection or peritonitis by local application of antiseptics or systemic antibiotics have not been sustainably successful. This has given rise to technologies that modify the catheter surface to reduce bacterial colonization and biofilm formation. An obvious choice was silver, in view of its widely recognized antimicrobial properties. Various technologies have been used to apply silver as a coating, such as ion-beam deposition and chemical bonding. While some studies have reported encouraging *in vitro* results, clinical studies have been disappointing. Bong et al. [27] using an iontophoretic silver coated central venous catheter found no significant difference in infection rates between these and plain catheters in a clinical trial. Crabtree et al. [28] used a PD catheter with silver applied by ion beam deposition, but again their clinical study showed no benefit over plain catheters. Antibiotics have also been used in coatings. Finelli et al. [29] coated silicone coupons with a hydrogel-liposomal preparation containing ciprofloxacin and inserted them into rats intraperitoneally, followed by a bacterial challenge at the same procedure. They found that coated catheters did not become colonized and the rats did not develop peritonitis, contrary to the findings in the plain catheters. However, this study does not give any indication of sustained activity beyond the first few hours. Another finding was that the coated catheters became covered by omentum, and omentectomy was necessary, suggesting unsatisfactory biocompatibility.

Coatings are commonly employed to protect biomaterials and devices from microbial colonization, and they often give encouraging *in vitro* results, but they almost invariably fail in clinical testing. One reason is that they rapidly become covered by host-derived conditioning film that obscures their activity. In the case of silver, the antimicrobial activity is dependent on silver ion release, and silver ions are very susceptible to inactivation by protein and chloride, both of which are abundant in the mammalian body. Another failing, especially in implants that require a long duration of activity, is that coatings are rapidly depleted by fluid flow. They must also be able to be applied on both outer and inner surfaces of catheters to be effective, and some spray-coatings do not reliably coat inner surfaces.

### 7.3. Catheter impregnation with antimicrobials

These considerations have stimulated interest in other antimicrobial technologies. Using an in-house technique, Kim et al. [30] impregnated silicone PD catheters with a mixture of chlorhexidine, silver-sulfadiazine, and triclosan, and showed that these had more than 10 days’ activity against staphylococci in vitro. They placed the PD catheters in rats and gave a single *S. aureus* bacterial challenge dose at insertion. While all the plain catheters became colonized, none of the impregnated ones did so after 7 days. While these results appear promising, again the only challenge took place at the point of catheter insertion, and no implications can be drawn regarding their ability to protect the PD catheters from further bacterial challenge during longer term use as duration of activity was not determined except by a zone plate method.

A group headed by Darouiche and Raad have published several papers showing that their catheters impregnated with rifampicin and minocycline can give sustained protection against catheter colonization in vivo [31,32]. The technique has been applied to central venous catheters and to external ventricular drainage catheters with clinical success [33]. Their group also showed that hemodialysis catheters impregnated with the two drugs were able to reduce infection rates significantly in a clinical study [34], and though they did not apply their technology to peritoneal dialysis catheters, it seems likely that it would be beneficial.

In 1989, we developed a new technology for impregnation of silicone devices [35]. The aims were to be able to process any silicone device post-manufacture, without changing its mechanical properties and conferring a long-lasting antimicrobial effect without increasing the risk of antimicrobial resistance. The principles of this technology were that certain chemically compatible antimicrobial agents could be evenly dispersed as molecules rather than particles throughout the silicone matrix; that they could migrate freely through the matrix in the absence of water; that extremely small quantities of the drugs would be released into the tissues; that the surfaces of the device would be protected over long periods from bacterial colonization and biofilm development; and that this would not give rise to resistant organisms. The aims were achieved in vitro using very rigorous clinically predictive tests. Protection from microbial resistance was achieved by application of the Dual Drug Principle [36–39]. We have shown that the process does not prevent bacterial attachment but kills all attached bacteria rapidly [40]. The protective activity is maintained for at least 28 days [41] and can prevent colonization of neurosurgical shunt catheters by *Staphylococcus epidermidis*, *Staphylococcus aureus,* and *Cutibacterium acnes* [42].

The testing protocols for the technology include serial plate transfer tests (SPTT) where segments of impregnated catheter are placed on seeded agar plates, and after incubation the zone of inhibition is measured. The segments are then transferred to a fresh seeded plate, and the process repeated until no zones are seen [35]. The SPTT should be seen as a screening procedure to establish activity extending beyond the 1–2 days that can result from just surface activity. A second assay is then performed to determine the time taken to kill all attached bacteria (tK100). Segments of impregnated catheter are exposed to suspensions of bacteria for 1 hour to allow attachment to take place. The segments are then incubated in fresh culture medium, and triplicate samples are removed daily for 3 days. The samples are sonicated, and viable bacteria are counted. Viable counts of attached bacteria fall from 5 log/mL to zero in 50 hours, after which no viable bacteria can be detected on prolonged culture [40]. Finally, a definitive test in flow conditions is carried out. Whole impregnated catheters are inserted into a custom-made rig that maintains temperature and humidity of catheters while culture medium is pumped through the catheters at 20 mL/hr to simulate cerebrospinal fluid (CSF) flow rate. Catheters are challenged with 1 mL of 10^8^ colony-forming units of bacteria on days 1, 14 and 28 of perfusion. Samples of effluent are taken for viable counting daily for 14 days after each challenge. This flow challenge assay showed that the impregnated catheters were able to withstand high numbers of bacterial challenge for at least 28 days of continuous perfusion [35,41]. Several clinical studies have shown the efficacy and effectiveness of the impregnated shunts, and the application of the technology to external ventricular drain catheters, and the cost savings resulting from reduced infection rates have been considerable [43–45]. A recent randomized controlled trial comparing plain, silver-coated, and antibiotic impregnated ventriculoperitoneal shunt catheters has shown that the impregnated catheter give a clinically and statistically significant reduction in infection, while the silver-coated catheter had no effect [46]. The impregnated catheter is now the standard of care in U.K. and U.S.A. A study of external ventricular drainage showed that the impregnated catheters could reduce infection rates while avoiding the use of systemic antibiotics, reducing antimicrobial resistance and *Clostridioides difficile* infection [47].

We have now extended the technology to urinary catheters and PD catheters using a modified formulation to address both the spectrum of pathogens and the need for longer duration of action. Using the same technology for impregnation, we have produced catheters containing rifampicin, sparfloxacin, and triclosan. The formulation is designed to be effective against the urinary and PD pathogens and to give a duration of protection of up to 3 months. The impregnated urinary catheter has been shown to reduce mineral deposition and to prevent colonization by multidrug-resistant gram-positive and gram-negative bacteria for approximately 3 months [48]. We have inserted the catheter into 40 patients in a safety and tolerability study, and there were no adverse events, the study catheter being preferred by most patients [49]. A randomized controlled trial of the promising impregnated urinary catheter has been seriously delayed due to disruption to regulatory bodies and supply routes arising from the political separation of the United Kingdom from Europe (BREXIT).

Based on the preliminary studies described above, we have embarked on a safety and tolerability study of a PD catheter with the same formulation as the urinary catheters. Previous studies suggest that this catheter will be able to reduce both exit site infection and peritonitis by the usual gram-positive and gram-negative PD pathogens, including multi-drug resistant gram-negative bacteria and MRSA. Though the protective activity of the catheter is unlikely to extend beyond 3 months, there is good evidence that if an infection-free period can be maintained for this long after catheter insertion, the risk of subsequent infection will be reduced. In one large retrospective study, patients who developed infection early had a higher risk of mortality. Also, early onset of peritonitis was associated with an overall higher rate of peritonitis [50].

The problems associated with commercialization, regulatory approval, and clinical acceptance of new devices are considerable, and arguably have been worsened by industrial downturns and BREXIT. The costs involved for companies in taking a new device from the laboratory to clinical application are becoming prohibitive, even when clear cost savings to health-care systems can be demonstrated. Regulatory test requirements are not always in line with clinical needs and are often needlessly costly as well as being uninformative and are ripe for radical revision [51].

## 8. Conclusion

Considerable progress has been made in developing a technology for impregnating silicone PD catheters with antimicrobials to reduce the risk of infection. Application of the same technology to neurosurgical shunt catheters provides evidence of the clinical efficacy of this approach and preliminary studies of its use in urinary catheters are also promising. A study of safety and tolerability of antimicrobial impregnated PD catheters is in preparation and will enable a subsequent randomized trial. Proven efficacy will provide a novel approach to reduce exit site infections and peritonitis to improve patient quality of life and reduce technique failure.

## 9. Expert opinion

The proven efficacy of antimicrobial impregnated catheters for the reduction of infections associated with the use of ventricular shunts in neurosurgery and their adoption as standard of care, provides strong evidence to support the use of a similar approach for producing catheters that will reduce the risk of peritoneal dialysis (PD) associated infections. This is urgently needed because infections are arguably the single most important complication limiting more widespread use of this valuable form of home-based dialysis. The current approach of training patients to maintain rigorous aseptic technique when performing fluid exchanges is insufficient to prevent all infections.

A major advantage of antimicrobial impregnated catheters is that they could be introduced rapidly into clinical practice because this would require no change to clinical pathways – the antimicrobial catheter simply replaces a conventional catheter. There will likely be a modest increase in the cost of the catheters, but this will be offset by a reduction in the costs of treating PD-associated infection, which are substantial.

An initial study of patient acceptability and safety is planned, though currently delayed by regulatory barriers. Once this has been conducted, and assuming the results are supportive, a randomized trial will be warranted to provide evidence of efficacy and to establish the magnitude of the benefit to inform formal health economic evaluations. Further research should focus on the duration of benefit from the antimicrobials impregnated into the catheters. The antimicrobials gradually diffuse out of the catheter over time and will eventually disappear completely. This is particularly relevant for PD catheters, which may remain in place for several years. One aspect to consider is that early prevention of bacterial colonization of the catheters may prevent biofilm formation, which may have long-term benefits even after antimicrobials have been lost from the catheters. Clinical studies will also monitor the emergence of bacterial antibiotic resistance, deemed unlikely because in choosing the combination of antimicrobials the ‘Dual Drug Principle’ was adhered to.

Future research into preventing PD-related infections may focus on modification of the catheter surface to further inhibit colonization and biofilm formation. It seems likely that this approach could be used in combination with antimicrobial impregnation to optimally minimize the risk of infection. Additionally, research is required on the best method to prevent migration of skin bacteria into the subcutaneous catheter tunnel.

The ultimate goal should be to develop PD catheters that form an effective barrier to colonization and infection, thereby improving the time for which PD remains effective in individuals, avoiding harm, and contributing to improved quality of life, as well as increasing the number of people who can benefit from PD.

## Article highlights

- Peritoneal dialysis is a widely used dialysis modality but is associated with a risk of potentially serious infections including exit site infection, catheter tunnel infection, and peritonitis
- Despite universal training of patients in the use of aseptic technique during fluid exchanges, Infections remain the most common and dangerous complication of peritoneal dialysis
- A novel technique for the impregnation of silicone devices with antimicrobial agents has been used to produce antimicrobial impregnated ventricular shunt catheters used in neurosurgery with proven clinical efficacy
- The same technology has been used to develop urinary and peritoneal dialysis catheters impregnated with sparfloxacin, triclosan, and rifampicin.
- Safety and tolerability have been demonstrated in urinary catheters, and a similar study is planned for peritoneal dialysis catheters.
